# The Mississippi Valley Medical Association

**Published:** 1892-11

**Authors:** 


BUFFALO MEDICAL AND SURGICAL JOURNAL
A MONTHLY REVIEW OF MEDICINE AND SURGERY.

*EDITORS:*
THOMAS LOTHROP, M. D. - - WM. WARREN POTTER, M. D.
All communications, whether of a literary or business character, should be addressed to the managing editor: 284 Franklin Street, Buffalo, N. Y.

Vol. XXXII. NOVEMBER, 1892. No. 4.

### THE MISSISSIPPI VALLEY MEDICAL ASSOCIATION.

The eighteenth annual meeting of this Association was held in Cincinnati, October 12–14, 1892, under the presidency of Dr. Charles A. L. Reed. As might be expected from the conjunction of two facts in the foregoing sentence—namely, the location and the president—the meeting was a successful, interesting and instructive gathering of representative physicians. The committee of arrangements, consisting of five delegates from each of the four medical societies of Cincinnati, with Dr. J. A. Fackler as Chairman and Dr. T. V. Fitzpatrick as Secretary, performed its functions in a most satisfactory manner. The discipline of such a committee, made up of delegates from the representative medical societies of a large city, and headed by competent officers, cannot be excelled, and other cities may well model committees, with similar duties, after the fashion adopted in Cincinnati.

The place designated for the meetings of the sections, both medical and surgical, were centrally located and contiguous to each other, which is of prime importance to the comfort of visitors and members.

One of the reasons that contributes to the success of the meetings of this association is the season of the year in which they are held. Mid-October in the Mississippi or Ohio Valleys shows the weather and the region at their best. Nothing could be more lovely than a ride through Southern Ohio, Indiana, Illinois, or Missouri, returning through Tennessee and Kentucky, at this season. The ever-changing hues and bright parti-colors of the foliage, together with the ripened harvest and autumnal grasses, present a picture pleasing to the eye and stimulating to thought.

The address on Advances in Therapeutics, by Dr. H. A. Hare, of Philadelphia, was a most interesting and scholarly paper, that deserves to be carefully read by the entire profession. The *Therapeutic Gazette* for October 15th publishes it in full. The address on Surgery, promised by Dr. Hunter McGuire, was omitted, owing to the failure of the author to present an appearance. It would appear that the duty is well-nigh imperative to fulfill an appointment to address a professional body on a specific subject, sickness or dire calamity alone preventing.

The social part of the programme was well carried out, and consisted of a reception at the Art Museum, which is so famous, and a reception and banquet at the Grand Hotel on consecutive evenings. These, together with the numerous private dinners and luncheons, served to keep the guests busy out of regular meeting hours.

The officers chosen for ensuing year were : President, R. Stansburg Sutton, M. D., Pennsylvania; First Vice-President, W. N. Wishard, M. D., Indiana ; Second Vice-President, W. S. Christopher, M. D., Illinois ; Secretary, T. V. Fitzpatrick, M. D., Ohio ; Treasurer, A. M. Owen, M. D., Indiana ; Chairman Committee of Arrangements, F. C. Woodburn, M. D., Indiana ; Committee on Credentials, C. S. Bond, M. D., Chairman, Indiana ; I. N. Love, M. D., Missouri ; J. B. Murphy, M. D., Illinois ; T. H. Stuckey, M. D., Kentucky ; Judicial Council, Theo. Potter, M. D., Indiana ; Chas. A. L. Reed, M. D., Ohio ; C. H. Hughes, M. D., Missouri ; and W. H. Daly, M. D., Pennsylvania.

Place of next meeting, Indianapolis, Ind.

Finally, the Association distinguished itself by daring to do the right thing with reference to the so-called code issue. Heretofore the rule has been, according to Article 3 of the Constitution of the Mississippi Valley Medical Association, that "membership in this Association shall be limited to those members in the profession of medicine who acknowledge an allegiance to the American Medical Association by signing its code of ethics." Notwithstanding this specific declaration of loyalty, the American Medical Association refused to admit delegates from the Mississippi Valley Medical Association at the Detroit meeting last June. In return for this singular act of courtesy, the Mississippi Valley Association changed the foregoing article to read, in effect, as follows : "Membership in this Association shall be limited to members of the medical profession in good standing." This is the sum of the whole question, and should be all the ethical declaration needed by any society. We have refrained from a discussion of this subject during the past several months, while it has been such a lively theme in the journalistic world, and we only allude to it now to show the manliness of the Mississippi Valley Medical Association in taking hold of this subject in the right way, and handling it in the proper spirit. The Medical Society of the State of New York shakes hands with the Mississippi Valley Medical Association, and meets it on common ethical grounds.

